# Urban-rural disparities in receipt of treatment for obstetric complications in Ethiopia: A multivariate decomposition analysis

**DOI:** 10.1371/journal.pone.0355684

**Published:** 2026-08-12

**Authors:** Asebe Hagos, Melak Jejaw, Tesfahun Zemene Tafere, Kaleb Assegid Demissie, Misganaw Guadie Tiruneh, Getachew Teshale, Andualem Yalew Aschalew, Nebebe Demis Baykemagn, Jenberu Mekurianew Kelkay, Azmeraw Tadele

**Affiliations:** 1 Department of Health Systems and Policy, Institute of Public Health, College of Medicine and Health Sciences, University of Gondar, Gondar, Ethiopia; 2 Department of Health Informatics, Institute of Public Health, College of Medicine and Health Sciences, University of Gondar, Gondar, Ethiopia; 3 Department of Public Health, College of Health Sciences, Debark University, Debark, Ethiopia; 4 Department of Medical Nursing, School of Nursing, College of Medicine and Health Sciences, University of Gondar, Gondar, Ethiopia; National Research Centre, EGYPT

## Abstract

**Background:**

Reducing disparities in access to emergency obstetric care is crucial for decreasing maternal morbidity and mortality, particularly in low-resource settings where inequitable access remains a major challenge. While systematic monitoring and analysis of these disparities are essential for developing effective interventions, urban–rural disparities in Ethiopia remain understudied. Therefore, this study aimed to assess urban-rural disparities and identify factors contributing to disparities in treatment receipt for obstetric complications among postpartum women six weeks after childbirth in Ethiopia.

**Methods:**

We analyzed nationally representative Performance Monitoring for Action (PMA) Ethiopia cohort data (2021–2023), including a weighted sample of 884 postpartum women who reported obstetric complications. Logit-based multivariate decomposition was used to quantify urban–rural disparities in the receipt of treatment and to identify contributing factors. Statistical significance was determined at p < 0.05, with 95% confidence intervals.

**Results:**

In Ethiopia, 61.4% (95% CI: 54.7–68.1%) of women received treatment for obstetric birth complications. Urban women had a higher treatment rate (80.1%) than rural women (55.3%), showing a 24.6 percentage point difference, indicating a high level of inequality. The decomposition analysis showed that endowment effects explained 153% of the disparity, while coefficient effects contributed –53.4%, indicating that differences in characteristics drive the gap, whereas differences in how these characteristics translate into treatment partially offset it. Significant endowment components explaining the disparity included: the 35−49 age category (−7.5%), Muslim religion (4.8%), rich wealth status (75%), birth preparedness discussions during antenatal care (1.9%), and facility delivery (73%). For the coefficient components, the significant contributing factors were: ages 25−34 years (26%), ages 35−49 years (24.4%), and having four or more ANC visits (−28.4%).

**Conclusion:**

Substantial urban-rural disparities in treatment receipt for obstetric complications exist in Ethiopia, driven by differences in both endowment and coefficient effect components. To address this gap, long-term strategies should address underlying socioeconomic disparities. In the short term, targeted interventions such as financial support for disadvantaged rural women, enhancing financial protection mechanisms, and improved maternal health services, particularly health facility delivery and birth preparedness initiatives, could narrow the disparity.

## Introduction

Reducing maternal mortality remains a global priority under the Sustainable Development Goals (SDGs), with countries striving to significantly address maternal deaths by 2030. SDG 3 targets an ambitious target: lowering the global maternal mortality ratio (MMR) to fewer than 70 deaths per 100,000 live births [1]. Between 2000 and 2022, MMR globally declined by 34.3%. Despite this progress, approximately 810 women still die every day from pregnancy and childbirth-related complications [2]. The burden is disproportionately high in sub-Saharan Africa, accounting for 70% of these deaths, while Southern Asia accounts for about 16% [2,3].

Ethiopia has achieved remarkable progress in reducing maternal mortality over the past decades. According to the United Nations Interagency Maternal Mortality Ratio estimates, the county’s MMR dropped significantly from 953 deaths per 100,000 live births in 2000–267 in 2020 [2,4]. However, in spite of these advancements, Ethiopia remains among the countries with the highest number of maternal deaths. In 2020 alone, approximately 10,000 maternal deaths were reported, accounting for 3.6% of the global maternal mortality [2]. Multiple studies have identified preventable obstetric complications such as hemorrhage, obstructed labor, pregnancy related hypertension, puerperal sepsis, and unsafe abortion as the leading causes of maternal mortality in Ethiopia [5–7]. These complications pose a particularly severe threat to women who deliver outside of health facilities, where access to emergency obstetric care is limited or unavailable.

The majority of maternal and newborn deaths occur during labor, delivery, and the immediate postpartum period [8]. Ensuring consistent and comprehensive maternal care particularly during childbirth and the immediate 24 hours after delivery is essential for reducing pregnancy related morbidity and mortality [9]. According to the World Health Organization (WHO), two key interventions can prevent 88–98% of maternal deaths caused by pregnancy related complications: timely access to high quality obstetric care, including facility-based deliveries attended by skilled health professionals, and the provision of quality emergency obstetric care [10,11]. Emergency obstetric care encompasses critical medical and surgical interventions intended to save the lives of mothers experiencing severe complications during pregnancy, childbirth, and the immediate postpartum period [10,12].

According to the 2021 PMA Ethiopia six-week postpartum national survey, 37.4% of women experienced at least one type of complication during or immediately after delivery. The most common self-reported complications were severe bleeding (20.2%), prolonged labor (16.2%), and convulsions (12.1%) [13]. Similarly, another study identified prolonged labor (23.4%) and hypertensive disorders (11.6%) as the most frequently reported complications [14]. While emergency obstetric care is critically needed, access remains limited for many women experiencing obstetric complications in low-resource settings [14]. In Ethiopia, for instance, the PMA survey revealed that although 78.1% of women with delivery-related complications received treatment or obstetric interventions, a substantial proportion still lacked access to these life-saving services [13]. Other studies have also consistently reported significant urban-rural disparities in maternal health service utilization in Ethiopia [15–19], with urban women having utilization rates that are more than 20 percentage points higher than those of rural women across key indicators [19]. These disparities are influenced by a range of demographic and socioeconomic factors, including maternal age, religion, household wealth index, educational level of women and their husbands, occupational status, media exposure, and knowledge of obstetric danger signs [20–23]. Additionally, utilization of ANC services, place of delivery, parity, and birth order have also been shown to contribute to these disparities [12,23,24].

The Ethiopian government has committed to reducing health inequalities as a main priority in its policy and national strategies [25,26]. To address these inequalities, systematic monitoring of existing disparities and rigorous analysis of their underlying determinants are essential prerequisites for developing targeted, evidence-based interventions [27]. While several studies have been conducted on inequalities in Ethiopia, most have primarily focused on wealth-based or socioeconomic inequalities in the utilization of maternal health services, such as ANC, skilled birth attendance, and postnatal care [15–17,28]. However, limited attention has been given to urban-rural disparities in receipt of treatment for emergency obstetric care. This study addresses that important research gap. Furthermore, it adds to the existing literature by applying multivariate decomposition analysis to identify the drivers of urban-rural disparities in receipt of emergency obstetric care.

Therefore, this study aimed to (1) quantify urban–rural disparities in receipt of treatment for obstetric complications during delivery or within 24 hours postpartum in Ethiopia, and (2) identify the contribution of individual sociodemographic, obstetric, and service-related factors to this disparity using multivariate decomposition analysis.

## Methods

### Study settings, design, and data sources

This study utilized data from the PMA Ethiopia project, which collects comprehensive reproductive, maternal, newborn, and child health indicators [29]. The PMA dataset is freely available from the Johns Hopkins Research Data Repository online archive (https://doi.org/10.34976/j5rf-8736). The dataset used to generate the findings of this study is included as a Supporting Information (S1 File). PMA Ethiopia conducted two longitudinal survey rounds: the first from 2019 to 2021 and the second from 2021 to 2023. In both cohorts, women were followed from pregnancy through one year postpartum, with data collected at four stages: baseline (during pregnancy), six weeks postpartum, six months postpartum, and one year postpartum [30].

For this analysis, we used data from the baseline and six-week postpartum surveys of the second cohort, as these datasets detailed maternal health information pertinent to our research objectives. The final dataset was constructed by merging the baseline and six-week postpartum datasets using unique participant identification numbers [31].

A multistage, stratified cluster sampling approach was used to select study participants [29]. In the first stage, four major regions of Ethiopia, Amhara, Oromia, Southern Nations, Nationalities, and Peoples’ Region (SNNP), and the Addis Ababa city administration were selected. Amhara, Oromia, and SNNP were further stratified into urban and rural areas, while Addis Ababa was considered a separate urban stratum. Using a sampling frame provided by the Central Statistical Agency (CSA), 162 enumeration areas (EAs) were selected with probability proportional to size within each stratum. In the final stage, 35 households were randomly selected from each EA [29].

### Study population and sample size

**Fig 1:**
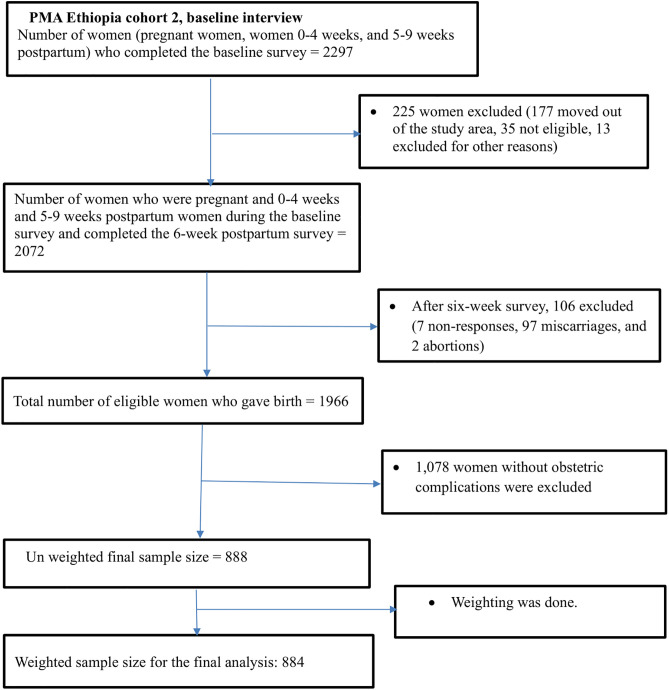
Flow chart of participant selection for analysis of urban-rural disparities in receipt of treatment for obstetric complications in Ethiopia, PMA-Ethiopia 2021-2023.

Women were eligible for inclusion in this study if they completed both the baseline survey and the six-week postpartum follow-up. In cohort two, 2,297 women completed baseline and 2,072 completed the six-week follow-up. After excluding women with abortion or miscarriage, 1,966 remained eligible [30,32]. Among these, 888 reported at least one obstetric complication at delivery or within 24 hours postpartum, corresponding to a weighted analytic sample of 884 women. The flowchart of participant selection is presented in Fig 1.

### Data collections

Data were collected electronically using the Open Data Kit platform on mobile tablet devices. To ensure the data quality, PMA Ethiopia implemented a comprehensive two-week training program for supervisors and resident enumerators (data collectors). The training covered survey protocols, questionnaire content, and interview techniques, followed by a three-day fieldwork simulation. Additional details are available in a referenced source [29].

The surveys captured essential reproductive, maternal, newborn, and child health indicators. The baseline survey gathered data on household socioeconomic characteristics, reproductive and fertility histories, and ANC visits [29,30]. During the six-week postpartum interview, additional maternal and newborn health indicators were collected. These included ANC (timing, frequency, and content), delivery services (facility-based delivery, skilled birth attendance, obstetric complication and treatment access), use of maternal waiting homes, and immediate postpartum care for mothers and newborns [29].

### Study variables and measurement

#### Outcome variable.

The outcome variable was receipt of any treatment for obstetric complication during delivery or within 24 hours postpartum. For analytical purposes, this variable was coded as 1 if a woman self-reported receiving treatment from any health facility (public, private, or NGO) for at least one obstetric complication (during delivery: heavy bleeding, leaking/rupture of membranes with no labor pain for >24 hours, absence of labor contractions >24 hours, preterm rupture of membranes, malposition, prolonged labor >12 hours, or convulsions; or within 24 hours postpartum: retained placenta, heavy bleeding, convulsions, or fever). The variable was coded as 0 if the woman did not receive treatment.

#### Equity stratifier variable.

The stratifying independent variable was the woman’s place of residence, categorized as a binary variable: urban = 1 and rural = 0.

#### Explanatory variables.

The explanatory variables in this study included the women’s age (15–24, 25–34, 35–49), marital status (single, married), education level of the mother (no education, primary education, secondary education, higher education), the respondent’s religion (Orthodox, Protestant, Muslim), household wealth index (poor, middle, rich), family size (<5, ≥ 5), intendedness of pregnancy (unintended, intended), parity (0, 1–2, 3–4, 5+), partner encouragement to attend ANC (no, yes), ANC visits (No ANC, 1–3 ANC, four or more ANC), early initiation of ANC (no, yes), discussed birth preparedness during ANC (no, yes), utilization of maternity waiting home (no, yes), place of delivery (home, health facility), and household food security status (food secure, mildly food insecure, moderately food insecure, severely food insecure).

Birth preparedness was assessed based on whether women discussed four essential topics with their healthcare provider during antenatal care visits: preferred delivery location, skilled birth attendance, transportation arrangements, and emergency care for danger signs. A positive response (“yes”) was recorded if all four topics were discussed, while a negative response (“no”) was assigned if any topic remained unaddressed [33]. The household wealth index was derived using principal component analysis from multiple household economic indicators, including ownership of household assets (such as electricity, television, radio, watch, telephone, refrigerator), vehicles, water/sanitation facilities, housing quality (construction materials), agricultural land, and livestock ownership [34,35].

Household food insecurity was assessed using a nine-item questionnaire (Q1-Q9) with follow-up frequency questions [36,37]. This tool evaluates respondents’ recall of food insufficiency experiences and related psychological distress over the previous 30 days. Each question includes a follow-up item assessing the frequency of occurrence, coded as: 3 (often), 2 (sometimes), 1 (rarely), or 0 (not at all). Based on these responses, households were categorized into four levels of food security status. A household was classified as food secure if it scored 0 or 1 on the first question (Q1) and 0 on questions Q2 to Q9. It was considered mildly food insecure if Q1 scored 2 or 3, or Q2 scored 1, 2, or 3, or Q3 scored 1, or Q4 scored 1, with Q5 to Q9 all scoring 0. A moderate level of food insecurity was defined when Q3 scored 2 or 3, or Q4 scored 2 or 3, or Q5 scored 1 or 2, or Q6 scored 1 or 2, while Q7 to Q9 scored 0. Finally, a household was categorized as severely food insecure if Q5 or Q6 scored 3, or if any of Q7, Q8, or Q9 scored 1, 2, or 3 [36–39].

### Statistical analysis

To account for the complex survey design, we applied sampling weights. Descriptive statistics such as mean with standard deviation, frequencies, percentages, and tables, were used to present the findings.

To assess disparities in receiving obstetric treatment for obstetric complication, we first estimated the rate of treatment for obstetric complication among urban and rural women. The observed mean difference between these groups was then decomposed into endowment and coefficient effect using multivariate decomposition analysis. This approach partitions the group difference in the mean, proportion, or other statistics into two key components: the endowment effect (E) and the coefficient effect (C). The endowment effects reflect how much of the urban–rural difference is due to different distributions of characteristics (e.g., wealth, education, facility delivery), whereas coefficient effects reflect differences in how these characteristics translate into treatment under urban versus rural conditions [40,41]. The contribution of each component is expressed as a percentage of the overall observed difference between groups in the decomposition analysis. These percentages may be negative or greater than 100%. A negative contribution suggests that the component suppresses or offsets the observed difference, whereas a contribution greater than 100% suggests that the component alone would predict a wider disparity than what is observed [40]. This usually happens when the overall disparity is reversed by the coefficient effect.

A logit-based multivariate decomposition analysis was employed, appropriate for the binary nature of the outcome variable. The analysis was conducted using the *mvdcmp* command in Stata, which supports various estimation methods for nonlinear models [40]. This command generates the mean difference between groups, along with the respective values for both the endowment and coefficient components. Additionally, it quantifies the contribution of each explanatory factor to the observed urban-rural disparity across both components. Prior to the decomposition, multicollinearity among independent variables was assessed. All data management and statistical analyses were performed using STATA V 17.

### Ethical approval and consent to participate

Ethical clearance was not required for this study, as we used secondary data from PMA Ethiopia, which is publicly available dataset from the Johns Hopkins Research Data Repository. The use of this secondary data adhered to ethical standards for the protection of sensitive health information. We ensured that the data were used exclusively for research purposes and that no personal identifiers were disclosed, maintaining confidentiality throughout the analysis. The original data collection procedure was conducted in accordance with the principles of the Helsinki Declaration.

## Results

### Background characteristics of study participants

In the final weighted sample, approximately 24.8% of women were resided in urban area, while 75.2% lived in rural areas. The mean age of the women was 26.9 ± 6.6 years (SD). Approximately 6.7% of urban residents and 26.3% of rural residents were followers of the Muslim religion. Among the study participants, about 2.9% of urban women and 29.3% of rural women reported having no formal education. Likewise, about 1.5% of urban residents and 37.6% of rural residents belonged to the poor wealth category.

Regarding the maternal health service utilization, 2.5% of urban residents and 14.5% of rural residents did not receive any ANC services during their pregnancy.

Among women who attend ANC visits, 15.5% of urban women and 64.9% of rural women failed to initiate ANC follow-up within the first 12 weeks of pregnancy. Similarly, 19.9% of urban and 63.2% of rural women did not discuss birth preparedness and complication readiness with health care providers during ANC visits. Home delivery was low among urban residents (1.8%), whereas 34.6% of rural women delivered at home. Additionally, severe household food insecurity was reported by 2.3% of urban household and 13.7% of rural households (Table 1).

**Table 1:** Characteristics of study participants and percentage of women received treatment for obstetric complications during delivery in Ethiopia, PMA Ethiopia, 2022 (N = 884).

Variables	Categories	Weighted frequency and percentage	Weighted frequency and percentage by place of residence		Received treatment for obstetric complications
			Urban	Rural	
Women age	15-24	342 (38.7%)	83 (24.3%)	259 (75.7%)	225 (65.8%)
	25-34	395 (44.7%)	111 (28.1%)	284 (71.9%)	236 (59.7%)
	35-49	147 (16.6)	25 (17%)	122 (83%)	83 (56.5%)
Marital status	Single	55 (6.2%)	16 (29%)	39 (71%)	33 (60%)
	Married	829 (93.8%)	203 (24.5%)	626 (75.5%)	511 (61.4%)
Respondents’ religion	Orthodox	331 (37.5%)	109 (32.9%)	222 (67.1%)	218 (65.9%)
	Protestant	261 (29.5%)	51 (19.5%)	210 (80.5%)	141 (54%)
	Muslim	292 (33%)	59 (20.2%)	233 (79.8%)	185 (63.4%)
Mother’s education level	No education	285 (32.2%)	26 (9.1%)	259 (90.9%)	143 (50.2%)
	Primary education	407 (46%)	86 (21.1%)	321 (78.9%)	242 (59.5%)
	Secondary education	132 (15%)	59 (44.7%)	73 (55.3%)	109 (82.6%)
	Higher education	60 (6.8%)	48 (80%)	12 (20%)	50 (83.3%)
Wealth status	Poor	346 (39.1%)	13 (3.8%)	333 (96.2%)	150 (43.4%)
	Middle	168 (19%)	9 (5.4%)	159 (94.6%)	100 (59.5%)
	Rich	370 (41.9%)	197 (53.2%)	173 (46.8%)	294 (79.5%)
Family size	< 5	455 (51.5%)	150 (33%)	305 (67%)	306 (67.3%)
	≥ 5	429 (48.5%)	69 (16.1%)	360 (83.9%)	237 (55.3%)
Intendedness of pregnancy	Unintended pregnancy	469 (53.1%)	105 (32%)	364 (68%)	262 (55.9%)
	Intended pregnancy	415 (46.9%)	114 (27.5%)	301 (72.5%)	281 (67.7%)
Parity	0	165 (18.7%)	56 (33.9%)	109 (66.1%)	131 (79.4%)
	1-2	356 (40.3%)	117 (32.9%)	239 (67.1%)	230 (64.6%)
	3-4	200 (22.6%)	32 (16%)	168 (84%)	111 (55.5%)
	5 +	163 (18.4%)	14 (8.6%)	149 (91.4%)	72 (44.2%)
Partner encouragement to attend ANC	No	176 (19.9%)	16 (9%)	160 (91%)	67 (38%)
	Yes	708 (80.1%)	203 (28.8%)	505 (71.2%)	476 (67.2%)
ANC visits	No ANC visits	153 (17.3%)	22 (14.4%)	131 (85.6%)	57 (37.3%)
	1 to 3 ANC visits	363 (41%)	56 (15.4%)	307 (84.6%)	212 (58.4%)
	Four or more ANC visits	368 (41.7%)	141 (38.3%)	227 (61.7%)	275 (74.7%)
Early initiation of ANC visit	No	711 (80.4%)	137 (19.3%)	574 (80.7%)	411 (57.8%)
	Yes	173 (19.6%)	82 (47.4%)	91 (52.6%)	133 (76.9%)
Discussed birth preparedness during ANC	No	735 (83.1%)	176 (23.9%)	559 (76.1%)	446 (60.7%)
	Yes	149 (16.9%)	43 (28.9%)	106 (71.1%)	7 (65.1%)
Maternity waiting home use	No	776 (87.8%)	182 (23.5)	594 (76.5%)	450 (58%)
	Yes	108 (12.2%)	37 (34.3)	71 (65.7%)	93 (86.1%)
Place of delivery	Home	322 (36.4%)	16 (5%)	306 (95%)	50 (15.5%)
	Health facility	562 (63.6%)	203 (36%)	359 (64%)	494 (88%)
Household food security status	Food secure	356 (40.3%)	105 (29.5%)	251 (70.5%)	244 (68.5%)
	Mildly food insecure	101 (11.4%)	28 (27.7%)	73 (72.3%)	62 (61.4%)
	Moderately food insecure	283 (32%)	63 (22.2%)	220 (77.8%)	168 (59.4%)
	Severely food insecure	144 (16.3%)	23 (16%)	121 (84%)	69 (47.9%)

### Urban-rural disparities in receiving treatment for obstetric complications

Treatment coverage was 61.4% overall, with a 24.6 percentage point gap between urban (80.1%) and rural (55.3%) women.

### Results of multivariable logistic regression analysis

Among urban women, those in the 25–34 age category (AOR = 2.38, 95% CI: 1.12–5.08) and the 35–49 age category (AOR = 5.85, 95% CI: 1.31–26.09) were more likely to receive treatment for obstetric complication compared to the 15–24 age group.

Parity was significantly associated with recipient of treatment for obstetric complications. Women with parity 1–2 (AOR = 0.27, 95% CI: 0.10–0.70) and parity 3–4 (AOR = 0.24, 95% CI: 0.06–0.96) had 73% and 76% lower odds of receiving treatment, respectively, relative the reference group.

Among rural women, having four or more ANC visits was associated with 2.47 times higher odds of receiving treatment for obstetric complication (AOR = 2.47, 95% CI: 1.03–5.87) compared to those with no ANC visits.

Health facility delivery was a strong predictor of receiving treatment for obstetric complications among both urban and rural women. Women who delivered at a health facility had 20-fold higher odds (AOR = 20.3, 95% CI: 6.01–68.68) of receiving treatment for obstetric complications among urban women, and 46-fold higher odds (AOR = 46.18, 95% CI: 24.60–86.70) among rural women (S1 Table).

### Results of multivariate decomposition analysis

Table 2 presents the results of the multivariate decomposition analysis, revealing a significant disparity in receiving treatment for obstetric complications between urban and rural residents. The decomposition showed that differences in characteristics (endowments) explained 153.4% of this gap, while coefficient effects contributed –53.4%. The negative contribution of the coefficient indicates that difference in behavioral or structural response among rural residents partially offsets the observed disparity. This suggests that if rural women had the same coefficient as urban women, the urban-rural disparity would be even larger.

**Table 2:** Detailed decomposition of receiving treatment for obstetric complications by place of residence among postpartum women in Ethiopia, using the PMA Ethiopia 2022 survey.

Decomposition	Coefficient with 95%CI		Percent explained		P-value		
Raw difference (R)	0.24679 (0.18915, 0.30444)		100		0.000		
Explained (E)	0.37853 (0.29391, 0.46315)		153.38		0.000		
Unexplained (C)	−0.13174 (−0.22584, −0.03764)		−53.38		0.006		
**Covariates**	**Category**	**Coefficient with 95%CI**	**Percent**	**P value**	**Coefficient with 95%CI**	**Percent**	**P value**
**Endowment (Explained characteristics) = Difference in characteristics (E)**					**Due to difference in coefficient (C)**		
Age	15 − 24	1	1		1	1	
	25 − 34	0.00842 (−0.00031, 0.01714)	3.41	0.059	0.06416 (0.00192, 0.12640)	26.00	**0.043**
	35 − 49	−0.01847 (−0.03543, −0.00151)	−7.48	**0.033**	0.06015 (0.01310, 0.10721)	24.37	**0.012**
Marital status	Single	1	1		1	1	
	Married	0.00093 (−0.00283, 0.00469)	0.38	0.627	−0.08797 (−0.32574,0.14981)	−35.64	0.468
Respondents’ religion	Orthodox	1	1		1	1	
	Protestant	0.00196 (−0.01050, 0.01442)	0.80	0.758	0.00932 (−0.03986, 0.05850)	3.78	0.710
	Muslim	0.01172 (0.00153, 0.02192)	4.75	**0.024**	−0.05057 (−0.10704, 0.00591)	−20.49	0.079
Mother’s education level	No education	1	1		1	1	
	Primary education	0.00055 (−0.01985, 0.02095)	0.22	0.958	0.03501 (−0.05747,0.12749)	14.19	0.458
	Secondary education	0.01224 (−0.03322, 0.05771)	4.96	0.598	0.00568 (−0.02437, 0.03574)	2.30	0.711
	Higher education	−0.00650 (−0.05868, 0.04568)	−2.63	0.807	−0.00113 (−0.00532, 0.00307)	−0.46	0.598
Wealth status	Poor	1	1		1	1	
	Middle	−0.00750 (−0.04879, 0.03379)	−3.04	0.722	0.00632 (−0.03693, 0.04957)	2.56	0.775
	Rich	0.18497 (0.06689, 0.30304)	74.95	**0.002**	0.05009 (−0.00187, 0.10206)	20.30	0.059
Family size	< 5	1	1		1	1	
	≥ 5	0.00849 (−0.01536, 0.03234)	3.44	0.485	−0.03710 (−0.10365,0.02944)	−15.03	0.274
Intendedness of pregnancy	Intended pregnancy	1	1		1	1	
	Unintended pregnancy	−0.00177 (−0.01128, 0.00774)	−0.72	0.715	−0.03454 (−0.09619, 0.02711)	−14.00	0.272
Parity	0	1	1		1	1	
	1 − 2	−0.03049 (−0.06682, 0.00584)	−12.35	0.100	−0.03893 (−0.11162, 0.03376)	−15.77	0.294
	3 − 4	0.01839 (−0.00517, 0.04195)	7.45	0.126	−0.04278 (−0.09910, 0.01353)	−17.33	0.136
	5 +	0.01505 (−0.02794, 0.05803)	6.10	0.493	−0.01570 (−0.07821, 0.04682)	−6.36	0.623
Partner encouragement to attend ANC	No	1	1		1	1	
	Yes	0.01919 (−0.00573, 0.04411)	7.77	0.131	0.07545 (−0.05838, 0.20928)	30.57	0.269
ANC visits	No ANC visits	1	1		1	1	
	1 to 3 ANC visits	−0.00255 (−0.03946, 0.03437)	−1.03	0.892	−0.03759 (−0.12023, 0.04504)	−15.23	0.373
	Four or more ANC visits	−0.01886 (−0.06752, 0.02980)	−7.64	0.448	−0.07019 (−0.14006, −0.00031)	−28.44	**0.049**
Initiation of ANC visits	Early initiation	1	1		1	1	
	Late initiation	−0.01540 (−0.04352, 0.01272)	−6.24	0.283	0.05703 (−0.07678, 0.19083)	23.11	0.404
Discussed birth preparedness during ANC	No	1	1		1	1	
	Yes	0.00478 (0.00042, 0.00914)	1.94	**0.032**	0.02168 (−0.00517, 0.04854)	8.79	0.114
Maternity waiting home use	No	1	1		1	1	
	Yes	−0.01540 (−0.04352, 0.01272)	−6.24	0.283	−0.00619 (−0.02456, 0.01217)	−2.51	0.509
Place of delivery	Home	1	1		1	1	
	Health facility	0.18039 (0.11542, 0.24536)	73.09	**0.000**	−0.07128 (−0.19068, 0.04813)	−28.88	0.242
Household food security status	Food secure	1	1		1	1	
	Mildly food insecure	0.00082 (−0.00288, 0.00452)	0.33	0.665	0.00765 (−0.01677, 0.03207)	3.10	0.539
	Moderately food insecure	0.00272 (−0.00265, 0.00809)	1.10	0.322	−0.00842 (−0.05646, 0.03962)	−3.41	0.731
	Severely food insecure	0.00729 (−0.00155, 0.01614)	2.96	0.106	−0.00208 (−0.02846, 0.02430)	−0.84	0.877

Furthermore, Table 2 outlines the individual contributions of each covariate to the urban-rural disparity in the rate of receiving treatment for obstetric complications within the endowment and coefficient components. The disparity in the endowment component was largely explained by household wealth status (74.95%), health facility delivery (73.09%), women aged 35−49 (−7.48%), affiliation with the Muslim religion (4.75%), and discussions about birth preparedness during ANC visits (1.94%). If the proportion of households with rich wealth status were equalized between urban and rural areas, the disparity in receiving treatment for obstetric complications would decrease by 74.95%. Similarly, if rural women had the same level of health facility deliveries as urban women, the urban–rural inequality in receiving treatment for obstetric complications would narrow by 73.09%. The age group of 35–49 years contributed −7.48% to the disparity, this indicating that this characteristic slightly narrowed the urban-rural gap. The distribution of women’s religion, particularly among Muslim religious followers, contributed to the widening of the disparity. This suggests that if rural women had the same proportion of Muslim religious followers as urban women, the urban-rural disparity would widen further by 4.75%.

Discussion on birth preparedness and complication readiness slightly contributed to the urban-rural gap in receipt of treatment. If rural women had engaged in birth preparedness and complication readiness discussions during their ANC visits at the same rate as urban women, the urban-rural disparity in obstetric complication treatment would have decreased by 1.94%.

The coefficient component reveals that differentials return to maternal age significantly widen the urban-rural gap in receipt of treatment. Women aged 25–34 and 35–49 receive a greater benefit from their age status than rural women in the same age group, contributing 26% and 24.3% respectively to the urban-rural disparity in obstetric complication treatment receipt.

The negative coefficient for having four or more ANC visits suggests that under current conditions, ANC is more protective for rural women than for urban women; if rural women derived the same returns from ANC as urban women, the urban–rural disparity in treatment would be larger.

## Discussion

Multivariate decomposition analysis is a valuable statistical approach for quantifying and explaining the factors that contribute to disparities in health care access and medical interventions between urban and rural residents [40,42]. It helps policymakers understand why disparities exist and explain the role of each factor in the urban-rural inequality. This study extends previous research on maternal health inequalities by focusing on treatment for obstetric complications and quantifying the specific contributions of socioeconomic, obstetric, and service-related factors to urban–rural disparities. The findings of this study revealed a statistically significant disparity in receiving of treatment for obstetric complication between urban and rural residents in Ethiopia. The difference between the two groups was 24.6 percentage points, which urban women are more likely to receive treatment for obstetric complication than rural residents. According to WHO, an absolute difference of 20 percentage points or more between the urban and rural residents constitutes a high level of inequality [43–45]. Therefore, this study indicates substantial inequality in receiving emergency obstetric care among women experiencing complications. These findings underscore the need for equity focused intervention to reduce urban-rural disparity in accessing and utilizing emergency obstetric care during childbirth and the postpartum period.

The study further revealed that observed differences in characteristics (endowment effects) accounted for 153.4% of the urban-rural disparity in receiving treatment for obstetric complications, while differences in coefficient effects contributed negatively (−53.4%) to the overall disparity. These findings align with previous studies on maternal health service utilization in Ethiopia and other sub-Saharan African countries, and these studies have demonstrated that urban-rural disparities in maternal health care access are largely attributable to differences in endowment characteristics [24,46]. These results suggest that while endowment-driven policies (targeting socioeconomic and healthcare access differences) could help address urban-rural disparities in obstetric complication treatment, merely equalizing endowment characteristics would be insufficient to eliminate the gap. Notably, even when rural women possess identical characteristics to their urban counterparts (including equivalent education levels), they still experience reduced access to obstetric treatment. The negative coefficient values indicate that differential returns to these characteristics actually reduce the observed disparity by 53.4%. This pattern may reflect underlying contextual differences, such as variations in healthcare access, service delivery, and socioeconomic factors that influence the receipt of treatment for obstetric complications [22]. Without these offsetting factors, the urban–rural treatment gap would be approximately 54% larger than currently observed. These findings highlight the need for complementary interventions that address endowment disparities and structural health system limitations to achieve equitable emergency obstetric care access.

This study identified household wealth status as a primary driver of the urban-rural disparity in receipt of treatment for obstetric complications. The analysis suggests that elevating rural women’s wealth status to match that of urban women with the highest wealth status would significantly reduce disparities in emergency obstetric care utilization. This result is consistent with previous studies conducted in Ethiopia [22], Tanzania [47], sub-Saharan African countries [20,46], and India [21], which have documented the significant role of the household wealth status in driving urban-rural disparities in maternal health service utilization.

Although Ethiopia provides free maternal health services, including emergency obstetric care, rural women still encounter significant financial obstacles in accessing treatment. The costs of transportation, temporary lodging and informal payments create substantial burdens for impoverished women in remote areas seeking care for obstetric complications during child birth [48,49]. Frequent ambulance shortages lead to reliance on costly private transport, which remains unaffordable for most poor women [50]. These challenges are amplified by geographical barriers including mountainous topography, long distances to health facilities, and inadequate road networks that inhibit access for obstetric emergency care [12,51,52]. Additionally, wealth disparities contribute to unequal access through additional pathways. Poor rural women typically have lower educational attainment and consequently less awareness of obstetric danger signs and complications. They also frequently lack autonomy in healthcare decision-making, often needing permission from male partners or family elders before seeking care [48]. This study suggests that addressing the urban–rural disparity in access to emergency obstetric care requires a holistic approach that includes improving women’s economic status, strengthening financial protection mechanisms, addressing transportation-related challenges, and enhancing health literacy among rural women regarding obstetric care [9,53–56].

Similar to wealth status, the place of delivery is a major factor contributing to the endowment component of urban-rural disparities in receiving treatment for obstetric complications. If rural women access to health facility deliveries at the same rate as urban women, the urban-rural gap in access to emergency obstetric care would be reduced by 73.1%. Women who deliver in health facilities are more likely to receive timely treatment for obstetric complications compared to those who give birth at home. However, health facility delivery rates in Ethiopia remain suboptimal, with a significant proportion of rural women lacking access to institutional delivery [17,18]. Evidence shows that one in two rural women delivers at home [17,57]. Our study underscores that increasing facility-based deliveries is a key strategy for reducing urban-rural disparities in access to emergency obstetric care for birth complications [23]. Given that wealth and place of delivery together account for nearly 150% of the disparity, interventions to enhance financial protection and promote facility-based births among rural women are likely to yield the largest reductions in inequality.

Older women aged 35–49 had a modest but significant role in reducing the urban-rural disparity in obstetric care access. If this age group in rural areas had not received treatment for obstetric complication at the same rate as their urban counterparts, the urban–rural disparity would have widened by 7.5%. This finding aligns with previous studies on the utilization of maternal health services [20]. This underscores the importance of equitable service provision for older mothers, particularly in rural settings, where advanced maternal age often coincides with higher obstetric risks [58].

Rural Muslim women may face significant barriers to accessing obstetric care during delivery complications compared to their urban counterparts due to multiple barriers [59,60]. In many rural Muslim communities, cultural and religious norms could discourage women from seeking care from male health providers, particularly for childbirth-related services, potentially leading to delays or avoidance of professional care [61,62]. Additionally, limited health literacy and lower autonomy in healthcare decision-making might further hinder access to treatment for obstetric complications [12].

The urban-rural disparity in obstetric care treatment was further influenced by differences in birth preparedness and complication readiness discussions during ANC visits. Equalizing rural-urban participation in these discussions may contribute to narrowing disparity by 1.94%. This aligns with established evidence linking birth preparedness to improved access to emergency obstetric care, as demonstrated in studies conducted in Ethiopia [63], Somaliland [64], and Bangladesh [65]. Our study underlines the need to strengthen birth preparedness and complication readiness counseling in rural ANC services to mitigate the urban-rural disparity in maternal healthcare access.

The coefficient component reveals that differentials effects of maternal age significantly widen the urban-rural gap in receiving treatment for obstetric complications. Urban-women aged 25–34 and 35–49 in urban areas experience disproportionately greater benefit from their age status compared to their rural counterparts, contributing 26% and 24.3% respectively to the urban-rural disparity in obstetric complication treatment receipt. In this study, middle-aged and older women were more likely to receive treatment for obstetric complications. This might be explained by the fact that older urban women often have better socioeconomic status compared to their rural counterparts, which could enable them to access maternal health services, including emergency obstetric care [66]. Furthermore, older women may possess greater autonomy and decision-making power regarding their health, allowing them to seek care more readily when complications arise [67,68].

The effect of four or more ANC visits (−28.4%) appears to contributes to narrowing the urban-rural gap in receiving treatment for obstetrics complication. A negative coefficient for ANC suggests that if rural women derived the same level of benefit from ANC services as urban women, the urban-rural disparity in receiving treatment for obstetric complications would widen by 28.4%. This implies that urban women may currently benefit less from ANC services, possibly due to their lower marginal effect or return. Although rural women may attend ANC visits, it is possible that the quality of services including counseling, follow-up, and provider engagement is lower in rural than urban settings, which could reduce the effectiveness of ANC in ensuring receipt of emergency obstetric care [69,70]. Additionally, rural women might face limited health literacy or cultural beliefs that discourage care-seeking even when complications occur. Structural challenges such as weak referral systems, geographical barriers, and shortages of skilled personnel may further weaken the relationship between ANC participation and treatment access for obstetric complication.

### Policy implication

To reduce the urban-rural disparity in receiving treatment for obstetric complications. The government and stakeholders need to implement both short and long term approaches [71,72]. In the short term, targeted interventions such as financial support programs for economically disadvantaged rural women and financial protection mechanisms could help narrow the disparity. Additionally, improving maternal health services, particularly health facility delivery rates and birth preparedness and complication readiness, strengthening referral systems and improving transport support are more immediately actionable which significantly reduce the urban-rural disparity in receiving treatment for obstetric complication [19,73]. Over the long term, structural equity policies are needed to address underlying socioeconomic differences between urban and rural populations, including education, wealth, and access to healthcare infrastructure, as well as systemic improvements to rural health facilities and workforce distribution [19,71]. By combining immediate service delivery interventions with longer-term structural strategies, Ethiopia can more effectively reduce urban–rural disparities in obstetric care and promote equitable maternal health outcomes [72].

### Strength and limitations of the study

The study utilized data from the most recent nationally representative survey conducted by PMA Ethiopia. Additionally, the survey was conducted among postpartum women at six weeks postpartum, which is a strength because it helps reduce recall bias compared to longer recall periods. Furthermore, the use of multivariate decomposition analysis is a methodological strength, as it allows for quantification of the extent to which urban-rural disparities are explained by differences in characteristics (endowment effect) versus difference in the effect of those characteristics (coefficient effects). However, the study also has several limitations that should be considered when interpreting the findings: First, findings pertain to women who reported obstetric complications and may not generalize to all births. Second, treatment was measured as a binary outcome, without capturing type, timeliness, or quality of care, which may mask more nuanced inequalities. Third, the study relied solely on self-reported complications without verification from medical records, which may introduce recall bias and subjectivity. The absence of clinical validation limits the accuracy and reliability of the findings. Future studies should consider linking survey data with clinical records or health facility data to validate self-reported complications and to capture more detailed measures of treatment quality and timeliness.

## Conclusion

There is a substantial urban–rural disparity in treatment receipt for obstetric complications during delivery or within 24 hours postpartum in Ethiopia. This disparity is largely driven by differences in wealth, place of delivery, age structure, religion, and birth preparedness, with additional contributions from differential returns to maternal age and ANC. To narrow this gap, the government and relevant stakeholders should address underlying socioeconomic inequalities, expand financial protection and targeted support for rural women, increase facility-based delivery and birth-preparedness counseling, and improve the quality of ANC and referral systems.

## Supporting information

S1 File Anonymized dataset underlying the analysis of urban-rural disparities in receipt of treatment for obstetric complications. (DTA)

S1 Table Factors associated with receiving treatment for obstetric complications during delivery among postpartum women, PMA Ethiopia, 2022 (N = 884). (DOCX)
