# Supplementary material for: Urban-rural disparities in receipt of treatment for obstetric complications in Ethiopia: A multivariate decomposition analysis
Source: PLoS One. 2026 Aug 12;21(8):e0355684. doi: 10.1371/journal.pone.0355684 (PMC13465970; doi:10.1371/journal.pone.0355684)
Supplement: Supplementary file 1 [file pone.0355684.s002.docx]

**S1 Table. Factors associated with receiving treatment for obstetric complications during delivery among postpartum women, PMA Ethiopia, 2022 (N=884).**

| Variables | Category | Urban | Rural |
| --- | --- | --- | --- |
|  |  | AOR with 95% CI | AOR with 95% CI |
| Women age | 15-24 | Ref | Ref |
|  | 25-34 | 2.38^*^ (1.12, 5.08) | 0.68 (0.30, 1.52) |
|  | 35-49 | 5.85^*^ (1.31, 26.09) | 0.60 (0.19, 1.81) |
| Marital status | Single | Ref | Ref |
|  | Married | 1.05 (0.30, 3.68) | 1.72 (0.51, 5.73) |
| Respondents’ religion | Orthodox | Ref | Ref |
|  | Protestant | 1.19 (0.50, 2.82) | 0.68 (0.33, 1.37) |
|  | Muslim | 0.54 (0.25, 1.18) | 1.54 (0.74, 3.18) |
| Mother's education  level | No education | Ref | Ref |
|  | Primary education | 0.75 (0.23 2.38) | 0.60 (0.31,1.16) |
|  | Secondary and above education | 1.11 (0.31, 3.91) | 1.31 (0.44, 3.92) |
|  | Higher education | 0.63 (0.17, 2.24) | 1.31 (0.12, 14.33) |
| Wealth status | Poor wealth status | Ref | Ref |
|  | Middle wealth status | 1.12 (0.04, 26.70) | 0.99 (0.49, 1.98) |
|  | Rich wealth status | 4.24 (0.23, 77.34) | 0.99 (0.44, 2.21) |
| Family size | Less than five | Ref | Ref |
|  | Five or more | 0.73 (0.32, 1.66) | 1.42 (0.67, 3.01) |
| Intendedness of pregnancy | Unintended | Ref | Ref |
|  | Intended | 0.60 (0.29, 1.20) | 1.50 (0.83, 2.72) |
| Antenatal care visits | No ANC visits | Ref | Ref |
|  | 1 -3 ANC visits | 0.71 (0.23, 2.165) | 1.62 (0.74, 3.53) |
|  | 4 + visits | 0.70 (0.24, 2.01) | 2.47^*^ (1.03, 5.87) |
| Timing of ANC | Early initiation | Ref | Ref |
|  | Late initiation | 1.36 (0.66, 2.83) | 0.97 (0.39, 2.41) |
| Parity | 0 | Ref | Ref |
|  | 1-2 | 0.27^**^ (0.10, 0.70) | 1.00 (0.39, 2.57) |
|  | 3-4 | 0.24^*^ (0.06, 0.96) | 1.23 (0.35, 4.22) |
|  | 5+ | 0.24 (0.05, 4.36) | 0.88 (0.20, 3.76) |
| Partner encouragement | No | Ref | Ref |
|  | Yes | 2.11 (0.56, 6.27) | 1.03 (0.52, 2.06) |
| Discussed birth preparedness during ANC visits | No | Ref | Ref |
|  | Yes | 1.87 (0.85, 5.26) | 0.94 (0.42, 2.12) |
| Maternal waiting home use | No | Ref | Ref |
|  | Yes | 1.37 (0.56, 3.35) | 1.95 (0.63, 6.02) |
| Health facility delivery | No | Ref | Ref |
|  | Yes | 20.32^***^ (6.01, 68.68) | 46.18^***^ (24.60, 86.70) |
| Food security status | Food secure | Ref | Ref |
|  | Mildly food insecure | 1.31 (0.47, 3.66) | 0.82 (0.32, 2.08) |
|  | Moderately food insecure | 0.60 (0.28, 1.28) | 0.77 (0.38, 1.55) |
|  | Severely food insecure | 0.65 (0.22, 1.88) | 0.57 (0.25, 1.29) |

Note: Parity was measured at baseline during pregnancy and reflects the woman's parity at the time of enrollment. Therefore, women classified as having parity 0 were nulliparous at baseline and have given birth during the follow-up period.
